# A Systematic Review of the Optimal Management of Pediatric Distal Radius Displacement Fractures: Open Reduction and Internal Fixation Versus Cast Placement

**DOI:** 10.7759/cureus.66696

**Published:** 2024-08-12

**Authors:** Brandon Krumbach, Christopher R Meretsky, Andreas Polychronis, Anthony T Schiuma

**Affiliations:** 1 Anatomy, St. George's University, True Blue, GRD; 2 Surgery, St. George's University School of Medicine, Great River, USA; 3 General Surgery, St. George's University School of Medicine, Great River, USA; 4 Orthopedic Surgery, Holy Cross Hospital, Fort Lauderdale, USA

**Keywords:** cast placement, open reduction and internal fixation (orif), distal radius displacement fractures (drdf), children, pediatric

## Abstract

Distal radius fractures are among the most common pediatric injuries, affecting thousands of children each year. These fractures often require clinical intervention to reduce displacement and ensure the proper healing of the growth plate and wrist bone. The primary objective of this comprehensive analysis is to compare the effectiveness of open reduction and internal fixation (ORIF) versus cast placement in the treatment of pediatric distal radius fractures, with the aim of identifying the optimal treatment approach. Therefore, a systematic review following the Preferred Reporting Items for Systematic Reviews and Meta-Analyses (PRISMA) guidelines was conducted on pediatric distal radius displacement fractures using extensive database searches from 2000 to 2024 for specific keywords, ensuring transparency and reproducibility. Our findings indicate that higher displacement necessitates ORIF to minimize long-term complications and ensure better functional outcomes for pediatric patients. Rare studies comparing ORIF and cast placement are analyzed, emphasizing the advantages and limitations of each approach. The document concludes that the choice between ORIF and casting depends on factors such as fracture severity, patient's age, and specific characteristics of the injury to ensure optimal outcomes in pediatric distal radius fracture management. In conclusion, our data suggests that ORIF and cast placement each have pros and cons for pediatric distal radius fractures, with the best treatment depending on fracture specifics and patient factors, but neither method is clearly superior for long-term outcomes.

## Introduction and background

Distal radius fractures (DRFs), occurring at the end of the radius bone near the wrist, are among the most common types of bone fractures. The radius is one of the two forearm bones, situated on the thumb side. The section of the radius that connects to the wrist joint is known as the distal radius. A fracture occurring in this area is referred to as a DRF [1]. The distal radius is the most frequent location for fractures in children and teenagers, accounting for 23-31% of all pediatric fractures [2]. The high incidence of these fractures is often attributed to falls or other accidents that involve an outstretched hand, a common scenario during children's active play and sports activities [3]. Children with DRF have typically been treated by performing a closed reduction procedure to realign the radius and restore its length [4].

Displacement or angulation can cause anxiety for families, and many factors are typically considered when determining the necessity of formal reduction. Questions include how much angulation and shortening are acceptable at various ages, whether the family and child will accept the deformity as it remodels, and if there will be any functional deficits for the child in the short or long term [5]. These concerns often result in procedural sedation for many of the approximately 280,000 DRFs seen annually in children under 10 years of age in the United States [6].

Optimal management of pediatric distal radius displacement fractures (DRDF) involves a multifaceted approach that prioritizes both immediate- and long-term outcomes. Initial assessment should include a thorough clinical examination and appropriate imaging, typically with X-rays, to determine the extent of displacement and involvement of the growth plate [7]. Closed reduction and casting remain the first-line treatment for most pediatric DRFs, given the remarkable healing capacity and remodeling potential in children [8]. However, the degree of displacement and the stability of the reduction must be carefully monitored. In cases where closed reduction is unsuccessful or the fracture is unstable, surgical intervention, such as percutaneous pinning or open reduction, may be necessary to ensure proper alignment and prevent long-term functional impairment. Follow-up care is crucial to monitor healing, assess for complications, and ensure that the child regains full function [9]. Rehabilitation, including physical therapy, may be required to restore strength and mobility [10]. The overall goal is to achieve anatomic alignment, promote optimal healing, and prevent future complications while minimizing the impact on the child's growth and development.

The primary goal of this study is to perform a thorough comparative evaluation of the most effective management techniques for pediatric DRDF. Specifically, the focus will be on comparing the success rates, outcomes, and potential complications of open reduction and internal fixation (ORIF) versus cast placement. The study intends to ascertain which treatment method yields superior results in terms of bone healing, functional recovery, and long-term prognosis for pediatric individuals. Through a meticulous analysis of clinical information, patient results, and current conventions, the research aims to provide evidence-backed recommendations to assist orthopedic surgeons in choosing the most suitable treatment strategy for young patients with DRFs.

## Review

Methods

Study Selection

**Figure 1:**
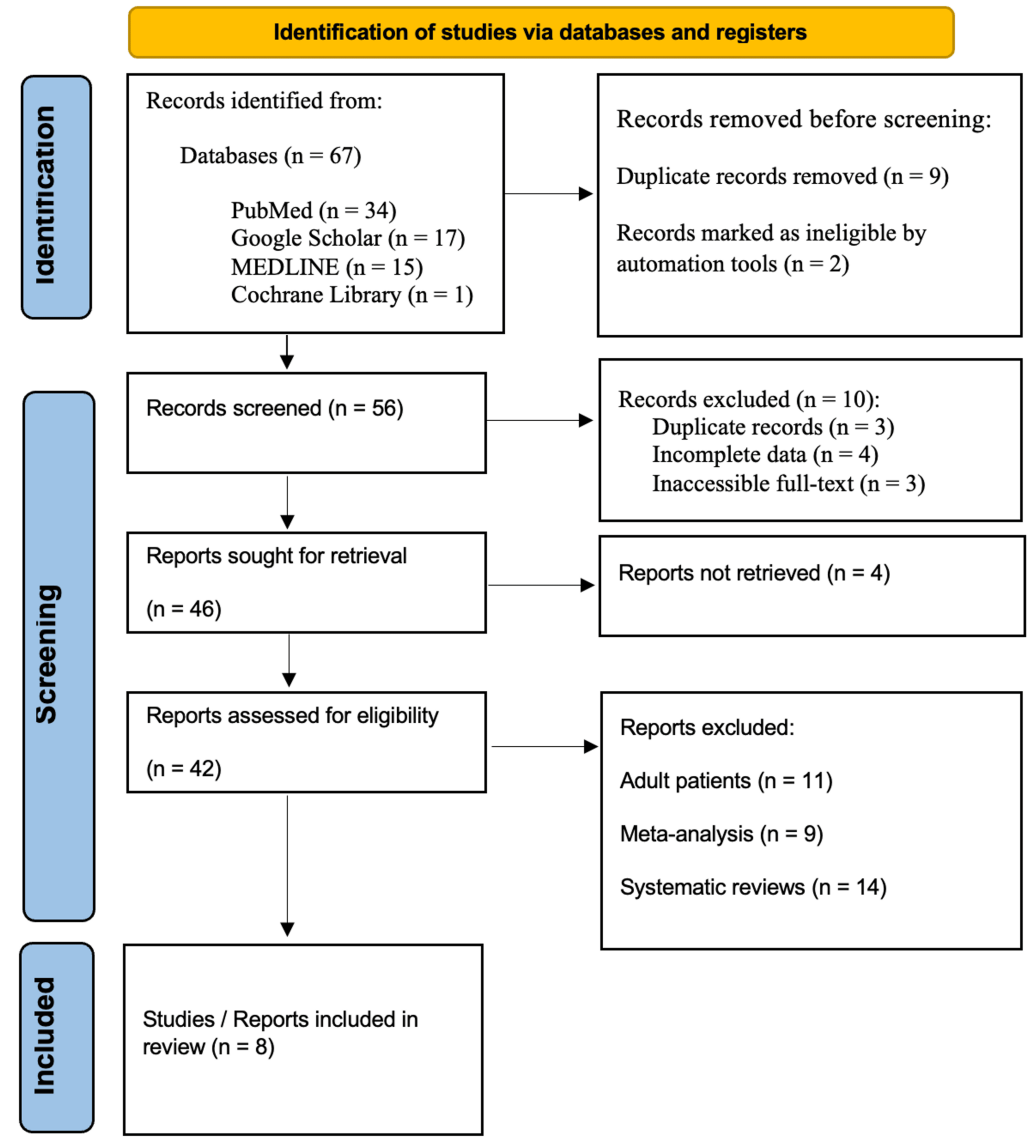
PRISMA flowchart: literature search and study selection n: number; PRISMA: Preferred Reporting Items for Systematic Reviews and Meta-Analyses Reference: [11]

Following the Preferred Reporting Items for Systematic Reviews and Meta-Analyses (PRISMA) guidelines, a systematic review was carried out by extensively searching databases such as PubMed, Google Scholar, MEDLINE, and the Cochrane Library for studies published from 2000 to 2024. Specific keywords like "Pediatric Distal Radius Displacement Fractures," "Pediatric Distal Radius Displacement Fractures and Open Reduction and Internal Fixation," "Pediatric Distal Radius Displacement Fractures and Cast Placement," "Distal Radius Displacement Fractures and children," and "Open Reduction and Internal Fixation and children" were used in the search. Adherence to the PRISMA guidelines ensured transparency and reproducibility in the review process (Figure 1).

Inclusion Criteria

The studies included in this review had to meet specific criteria. Firstly, they needed to involve human subjects undergoing large-scale pediatric DRDF. Secondly, they were required to report outcomes on various factors such as passive wrist range of motion, forearm rotation loss, deformity, and need for corrective surgery. Lastly, the studies had to be published in English.

Exclusion Criteria

However, we did exclude some studies from our selection. Studies that did not report adequate data specifically on pediatric DRDF cases were excluded. We also did not include meta-analyses, reviews, or editorials that lacked original findings. Research exclusively conducted in adults and other fracture types were also not considered. This discerning selection process served to reinforce the relevance and reliability of our review by focusing only on primary studies directly related to the human population of interest.

Outcome Measures

The length of hospital stay is a crucial outcome measure for evaluating the efficiency and effectiveness of different treatment modalities for pediatric DRF. This measure provides insights into the overall recovery process, resource utilization, and the impact of different treatments on hospital operations. A shorter hospital stay is generally indicative of a less invasive procedure and faster recovery. Postoperative pain is a significant concern in pediatric patients, as it can affect recovery, rehabilitation, and overall well-being. The quality of postoperative pain should be measured using standardized pain scales. Postoperative infection is a critical outcome measure, as it directly impacts patient safety, recovery, and treatment success. Monitoring infection rates provides valuable information on the safety and efficacy of the treatment methods used. High infection rates can indicate issues with surgical technique, postoperative care, or other related factors.

Results

Table 1 below provides an overview of the studies included in this review, which involve human subjects undergoing large-scale pediatric DRF. These studies compare two primary management approaches: ORIF and cast placement. The table analyzes various aspects of these studies, including the type of study and study design, the treatment approach used, the hypothesis proposed, the sample size and patients treated, parameters and safety considerations, and the main outcomes. First, the type of study and study design category details the methodology and design of each study, such as randomized controlled trials, cohort studies, or case-control studies. This information helps to understand the robustness and reliability of the study findings. Second, the treatment approach used provides specific details about the treatment modalities employed, focusing on ORIF and cast placement. This section highlights the differences in procedural techniques and approaches between the two treatment options. Third, the hypothesis proposed outlines the primary hypotheses or research questions that the studies aimed to address. Understanding the hypotheses helps to clarify the objectives and scope of each study. Fourth, the sample size and patients treated category details the number of participants included in each study and the demographics of the treated patients. This information is crucial for assessing the generalizability and applicability of the study findings to different populations. Fifth, parameters and safety considerations include key parameters measured, safety considerations taken into account, and criteria for evaluating the success and safety of the treatments. This section provides insights into how the studies ensured patient safety and measured treatment outcomes. Lastly, the main outcomes highlight the primary findings and conclusions of each study, emphasizing the effectiveness and safety of the treatment approaches. This category summarizes the evidence on the comparative efficacy of ORIF and cast placement in managing pediatric DRF. This comprehensive analysis allows for a detailed comparison of the efficacy and safety of ORIF and cast placement in managing pediatric DRDF, providing valuable insights for clinical decision-making.

**Table 1:** Studies (2000-2024) included in this review that involve human subjects undergoing large-scale pediatric distal radius displacement fractures and managed using ORIF and cast placement RCT: randomized controlled trial; CI: cast index; CR: closed reduction; DRF: distal radius fracture; LOR-TIKW: limited open reduction and transepiphyseal intramedullary fixation using Kirschner wire; TEN: titanium elastic intramedullary nails; ORIF-PS: open reduction and internal fixation and percutaneous surgery; LOS: length of stay; DMJ: diaphyseal metaphyseal junction; AEs: adverse events; SAEs: serious AEs; MAEs: minor AEs; PI: padding index; Cal: Canterbury index; GI: gap index; 3PI: three-point index; LOR: loss of reduction; UCLA: University of California at Los Angeles; MEPS: Mayo elbow performance score; CI: confidence interval; ORIF: open reduction and internal fixation References: [12-19]

Reference	Type study and study design	Treatment approach	Hypothesis	Size sample and participants' information	Parameters/safety considerations	Outcomes	
						Primary	Secondary
Laaksonen et al. (2021) [12]	RCT comparing casting in bayonet position to pin fixation	Traditionally treated with CR. Correction of shortening may not be necessary	Prospective cohort for non-participants, non-eligible cohort for enhancing external validity	60 patients under 11 years randomly assigned to casting or surgery groups	Potential AEs have been categorized as SAEs and MAEs. SAEs: Complications due to iatrogenic, procedural anesthesia, permanent nerve injury, systemic infections, and deep infection of the fracture site. MAEs: superficial infection, cast sore, non-union, implant failure, re-fracture, tendon injury, or nerve palsy	Total active forearm rotation ratio and total active wrist range of motion ratio at six months	Axial radiographic alignment, wrist extension, grip strength, forearm/hand length, patient-reported outcomes, and pain questionnaire. 0% of patients had visible deformity at follow-up. The mean dorsal angulation of radius was 2° between 2.5 and 4.5 years at follow-up
Pavone et al. (2020) [13]	Retrospective case-control study	Two attempts at CR, guided by fluoroscopy, were allowed before surgical intervention. Following surgery, all patients were immobilized with a long arm cast for a period of six weeks	Based on the risk factors, CR and casting represent the widely accepted primary treatment approach for DRFs	101 pediatric patients with DRF who received conservative treatment were classified into two groups: Group A (non-displaced) and Group B (with secondary displacement)	The radiographic assessment included the following: initial translation grade, initial reduction quality, and fracture status. Indices: CI, PI, CaI, GI, and 3PI	Group A: 37 (47.4%) of these patients achieved anatomic reduction, while 48.7% had fractures in both bones. Group B comprised 13 patients: Only three (13.0%) of these patients achieved anatomic reduction. A significant 65.2% of the group presented fractures in both bones	Casting is a simple, safe, and effective treatment for DRF in children. While conservative treatment is the gold standard for non-displaced fractures, it is also indicated for approximately 50% of displaced fractures
Abson et al. (2016) [14]	RCT comparing the surgeon seniority (resident vs. attending surgeon) with the CI and amount of displacement/angulation post-reduction	-	The extent of fracture re-displacement and the quality of cast molding were found to be linked to the experience level of the surgeon in managing displaced pediatric DRF that needed manipulation under anesthesia	143 pediatric patients with a DRF were classified into two groups: Group 1 (surgeon seniority) and Group 2 (CI and amount of displacement/angulation post-reduction)	-	There was no significant difference in CI for surgical performance between resident and attending surgeons based on experience level (P=0.14). Similarly, no difference in re-displacement rates was observed for fracture types relative to surgeon seniority	Residents seem well qualified in cast application
Rabinovich et al. (2021) [15]	RCT evaluating the effectiveness of CR and casting techniques for treating DRF and distal both-bone forearm fractures in pediatric patients	Simulated exercises involving reduction and casting of DRF. These simulations were conducted both at the beginning and at the conclusion of their six-month clinical rotation	Residents who received repeated simulation training throughout their rotation showed further improvement in their skills	28 residents treated a total of 159 DRFs and/or distal both-bone forearm fractures using CR and casting	Radiographic evaluations were performed to compare post-reduction fracture angulation, displacement, CI, and LOR	The post-simulation group demonstrated improved fracture reduction outcomes compared to the pre-simulation group, as evidenced by lower post-reduction radius angulation, lower maximal angulation, lower CI, and lower LOR	There was no statistically significant difference in radiographic parameters, CI, or LOR rates between residents
McLauchlan et al. (2002) [16]	RCT	Fractures were treated in two ways: either by realignment (manipulation) and a cast alone or with the addition of a thin metal pin (Kirschner wire) inserted through the skin	The traditional management of completely displaced fractures of the DR in children has been closed manipulation and casting	68 children with completely broken ends of their DR near the wrist	-	The K-wire group demonstrated significantly improved maintenance of reduction, resulting in a lower requirement for follow-up radiographs. No significant difference in clinical outcome was observed three months post-injury; the manipulation group exhibited a higher rate of secondary procedures	Seven out of 33 patients in the manipulation group required a second procedure due to unsatisfactory positioning, compared to zero out of 35 in the K-wire group
Georgiadis et al. (2020) [17]	Observational study on the treatment preferences for DRF among pediatric orthopedic surgeons and to assess whether their decision-making uncertainty was sufficient to consider randomizing treatment options	Respondents could select one of the following treatment options: (a) attempt anatomic reduction with sedation or (b) non-sedated immobilization	-	28 DRF scenarios in children aged 3-10 years were constructed in an electronic survey and received ORIF management	This analysis focused on patient factors (age, health status), fracture characteristics (severity, displacement), and surgeon experience to determine what influences treatment recommendations and a patient's willingness to be randomized in a treatment trial	Complete displacement was a predictor of decreased sedation. As coronal plane angulation increased, willingness to randomize decreased	A randomized, prospective trial comparing non-sedated immobilization with sedated/anesthetized reduction for the treatment of displaced pediatric DRFs
Wang et al. (2022) [18]	Observational study comparing the effectiveness of LOR-TIKW versus ORIF in the management of DRF	LOR-TIKW and ORIF-PS in treating irreducible DRF in older children	The hypothesis suggests that LOR-TIKW shows promise as a method that offers multiple advantages compared to ORIF-PS	26 children (aged 10-14 years) treated in our hospital for distal radius DMJ fractures with LOR-TIKW or ORIF-PS	Clinical, demographic, and radiographic information, treatment expenses, recovery duration, functional outcomes according to price criteria, complications, and postoperative angulation and displacement were analyzed for children treated using the two groups	The operation duration was reduced, the surgical incision size was smaller, the expense of internal fixation was decreased, and the healing period was shorter when utilizing LOR-TIKW	Postoperative fracture alignment showed a minor increase in angulation and a slightly higher displacement within the LOR-TIKW group
Kong et al. (2021) [19]	Observational study comparing the therapeutic efficacy of TEN versus ORIF in treating humeral fractures in children	The two groups were compared based on intraoperative bleeding, operation time, LOS, and fracture healing time	TEN is an effective technique for treating humeral fractures in children, offering several advantages	69 patients including 41 males and 28 females, ranged in age from six to 12 years old, with a median age of eight years	The two groups were compared based on intraoperative bleeding, operation time, LOS, and fracture healing time. The therapeutic effect was evaluated six months after the surgery using the shoulder range of motion, elbow range of motion, UCLA shoulder function score, and MEPS	In the TEN group, intraoperative bleeding, operation time, and fracture healing time were significantly less than in the ORIF group. There was no significant difference in the LOS between the two groups. The follow-up period was three and six months. The shoulder range of motion, elbow range of motion, UCLA shoulder function score, and MEPS were all greater in the TEN group compared to the ORIF group	The complication rate did not differ significantly between the two groups

According to the data in Table 1, the management of DRDF has been a subject of extensive research and debate, particularly when comparing ORIF with cast placement. ORIF is often favored for its ability to achieve precise anatomical alignment and stable fixation, which can facilitate early mobilization and potentially shorten hospital stays. This surgical approach is particularly advantageous in complex or severely displaced fractures where closed reduction and casting may fail to maintain satisfactory alignment. On the other hand, cast placement remains a widely used nonoperative method due to its non-invasiveness and lower immediate complication rates. However, cast placement can be associated with higher incidences of re-displacement and malunion, necessitating careful monitoring and possible subsequent interventions. Comparative studies have shown that while ORIF generally offers superior outcomes in terms of anatomical restoration and functional recovery, it also carries risks such as infection and hardware complications. Conversely, cast placement, though less invasive, may require longer immobilization periods and has a higher likelihood of requiring additional treatments. Ultimately, the choice between ORIF and cast placement should be individualized, considering factors such as fracture severity, patient age, and overall health, to ensure optimal outcomes in the management of pediatric DRDF.

Discussion

Due to rapid healing and the potential for remodeling residual deformities, nonoperative management is generally preferred for DRFs in pediatric patients. Once skeletal maturity is achieved, remodeling is no longer anticipated, and fractures are treated similarly to adult practices, often requiring surgical intervention. Although management guidelines for DRFs in both young and skeletally mature patients are well established, there remains controversy over the optimal treatment for patients nearing skeletal maturity [20]. Treating displaced, comminuted, intra-articular DRFs with closed methods, such as pins and plaster or external fixation, often results in unsatisfactory outcomes in most cases [21]. DRFs are among the most common fractures. For less displaced fractures, closed reduction and casting are the preferred treatment method. For more complex fractures, ORIF is used. ORIF helps restore the wrist's anatomy, facilitating quicker recovery and improving therapeutic outcomes [22].

This study aimed to compare clinical and functional outcomes of ORIF versus closed reduction and casting for displaced pediatric DRFs. The researchers hypothesize that while ORIF provides the advantage of anatomic restoration, casting may still be sufficient in many cases and avoids the risks of surgery. Understanding differences in complication rates, time to healing, and long-term function between the two approaches could help guide clinical decision-making for individual fractures. The results of this comparative analysis may provide evidence to determine the optimal management strategy.

ORIF

According to the outcomes of the current systematic review, ORIF offers several advantages over closed treatment for displaced pediatric DRFs. By surgically exposing the fracture site and realigning the bones directly, ORIF allows for the precise anatomical restoration of the articular surface and radial length. This can facilitate early range of motion exercises and prevent long-term post-traumatic arthritis. Inserting plates or screws also provides stable internal fixation, enabling faster healing compared to casting. However, ORIF also has some limitations. It carries risks of surgical complications like infection and nerve or blood vessel injury from extensive soft tissue dissection. Younger children may not tolerate general anesthesia as well. Implants also require later removal surgery. Perhaps most significantly, high-quality studies have not clearly shown ORIF to consistently produce superior long-term functional outcomes over closed treatment for all fracture patterns. Patient factors must be considered to determine if the benefits outweigh the risks in each individual case.

Ortega et al. retrospectively reviewed 16 children under the age of 13, with a total of 17 fractures of the radius, ulna, or both, who underwent ORIF. ORIF was performed in 14 cases where a closed reduction was deemed unacceptable and in three cases involving unstable open fractures of the radius. The average age of the patients was 9.4±2.3 years (range: 5.0-12.5). Among the 14 fractures with an unacceptable closed reduction, soft tissue interposition was encountered in seven cases. Fixation was achieved using plates and screws, percutaneous Steinmann pins, or intramedullary Steinmann pins. There were no delayed unions, nonunions, infections, or neurovascular injuries. With an average follow-up of 12.3 months, all 17 fractures had excellent outcomes, showing a forearm rotation loss of less than 10°. Their study suggests that excellent results can be achieved with ORIF in pediatric forearm fractures, with no increased risk of complications when the procedure is performed under proper indications [23,24]. 

According to Wright et al., utilizing ORIF with a volar fixed-angle implant led to secure fixation of the distal articular fragments, enabling early wrist movement after surgery. Both groups showed similar PRWE and DASH scores, but the ORIF group exhibited improved intra-articular step-off, volar tilt, and radial length. The ORIF procedure had minimal complications, did not require implant removal, and allowed for the early initiation of wrist range of motion post-surgery without reduction loss [25]. Obviously, ORIF has become essential in the management of fractures, especially when conservative treatments prove ineffective. In the context of pediatric DRDF, ORIF emerges as a crucial method. This review delves into the most effective approaches to managing these fractures in children, emphasizing the importance of ORIF supported by recent evidence and clinical guidelines. ORIF is typically indicated for pediatric DRFs that are unstable, irreducible, or associated with significant soft tissue injury [26]. It is also considered when closed reduction fails to achieve or maintain satisfactory alignment or when there is a high risk of malunion [3]. The goal of ORIF is to restore the anatomical alignment of the radius, ensuring stable fixation and promoting optimal functional recovery.

The data from the current systematic review highlights that ORIF is emerging as a prevalent treatment modality for managing DRFs in pediatric patients. In 2017, researchers examined treatment trends for pediatric DRFs in Korea by analyzing data from 2011 to 2015 provided by the Korean Health Insurance Review and Assessment Service. Using the International Classification of Diseases, 10th revision (ICD-10) codes and procedure codes, they identified patients under 18 years old with newly diagnosed DRFs. A total of 181,218 DRFs were recorded from 2011 to 2015. Most DRFs (92.2%) were managed conservatively. Surgical fixation was performed on 14,219 DRFs (7.8%), with the annual proportion of surgically treated DRFs remaining stable. Among those undergoing surgical fixation, closed pinning (9,664 DRFs, 68%) was the most common procedure. However, the use of ORIF increased steadily over time. Age-wise, the proportion of ORIF increased while closed pinning decreased as age increased. The researchers concluded that in Korea, most pediatric DRFs were managed conservatively, with closed pinning being the most popular surgical procedure. However, the proportion of ORIF has been steadily increasing each year [27]. Also, Goel et al. reported that ORIF with a buttress plate is an excellent treatment for displaced intra-articular DRFs, with approximately 91% of patients achieving excellent-to-good anatomical and functional outcomes [28].

Cast Placement

The analyzed outcomes of the present review reveal that cast placement has certain advantages over ORIF for displaced pediatric DRFs. As a closed treatment, it avoids the risks and costs associated with surgery. Younger children often tolerate casting better than anesthesia or inpatient procedures. Casting can still achieve and maintain acceptable alignment in some fracture patterns. It allows for bone healing through callus formation rather than relying on hardware. Cast changes can also allow the gradual correction of residual deformity. However, cast treatment has limitations as well. It provides less accurate anatomic reduction compared to direct visualization with open reduction. Healing in malunion can compromise function or require later corrective osteotomies. Immobilization in a cast also delays range of motion and rehabilitation. It may be difficult to reduce severely displaced or comminuted fractures without surgery. There is a risk of impairment or permanent deformity if reduction cannot be achieved or maintained closed.

Good initial reductions and proper casting techniques are necessary when treating distal radius and forearm fractures nonsurgically [29]. Achieving a good initial reduction of the fracture is crucial for successful nonsurgical treatment, and proper casting techniques are necessary to immobilize the fracture and maintain the reduction [30]. However, maintaining an acceptable reduction is not always possible, despite these efforts. Factors such as fracture pattern, displacement, and patient characteristics can make it difficult to keep the fracture aligned during the healing process [31]. In cases where the reduction cannot be maintained with casting alone, further intervention may be necessary, such as repeat reduction attempts, the use of percutaneous pins or other stabilization techniques, or even surgical treatment in some instances [32]. Careful monitoring of the fracture reduction during the course of treatment is essential. Regular radiographic assessments are necessary to ensure the fracture remains in an acceptable position as it heals. Close monitoring and a willingness to intervene when necessary are important to ensure the best possible outcomes for these patients [33].

Maintaining acceptable reduction is not always feasible, with re-displacement or re-angulation being the most commonly reported complications. These complications can be attributed to factors broadly categorized into three groups: fracture-related, surgeon-related, and patient-related [34]. Historically, the quality of casting has been assessed subjectively. To address this, various casting indices have been proposed by different authors, aiming for a more objective evaluation [35]. These indices include the cast index, padding index, gap index, three-point index, and second metacarpal-radius angle [36]. For DRFs, the three-point index is considered the most valuable measurement for predicting re-displacement among surgeon-related factors. However, this index has not been applied to forearm fractures, where the other indices appear more useful in predicting re-displacement. It is important to interpret casting indices in conjunction with fracture characteristics and patient factors, rather than as isolated metrics [37].

Cast treatment is less invasive and more cost-effective than surgical treatment. However, surgery is often the preferred option for this common type of DRF. Patients who have a non-acceptable position after closed reduction are more likely to benefit from surgery compared to those with an acceptable position post-reduction [38].

## Conclusions

ORIF and cast placement have their own advantages and disadvantages in treating displaced pediatric DRFs. ORIF allows for the direct visualization and restoration of the fracture site but comes with surgical risks and the need for eventual hardware removal. On the other hand, casting is a non-invasive option that avoids surgery-related complications but may result in less precise reduction and hinder rehabilitation progress. A major drawback of both methods is the lack of strong evidence supporting one approach over the other for achieving better long-term function. The best course of treatment is dependent on the specific characteristics of the fracture and the individual patient. For simpler fractures, casting may be sufficient, while more complex injuries with articular involvement could benefit more from ORIF. Younger children may be better off initially with casting to avoid the risks associated with anesthesia. However, there is a potential for deformity or impairment if the reduction cannot be achieved or maintained with closed treatment. Ultimately, a thorough evaluation of factors such as fracture severity, patient age, and orthopedic expertise is necessary to determine the most suitable technique for each clinical scenario. 
